# Alterations of the Intestinal Permeability are Reflected by Changes in the Urine Metabolome of Young Autistic Children: Preliminary Results

**DOI:** 10.3390/metabo12020104

**Published:** 2022-01-23

**Authors:** Cristina Piras, Michele Mussap, Antonio Noto, Andrea De Giacomo, Fernanda Cristofori, Martina Spada, Vassilios Fanos, Luigi Atzori, Ruggiero Francavilla

**Affiliations:** 1 Department of Biomedical Sciences, School of Medicine, University of Cagliari, 09124 Cagliari, Italy; cristina.piras@unica.it (C.P.); antonio.noto@unica.it (A.N.); martina.spada@unica.it (M.S.); latzori@unica.it (L.A.); 2 Department of Surgical Sciences, School of Medicine, University of Cagliari, 09124 Cagliari, Italy; vafanos@tiscali.it; 3 Child Neuropsychiatry Unit, Department of Basic Medical Sciences, Neuroscience and Sense Organs, University of Bari Aldo Moro, 70121 Bari, Italy; andrea.degiacomo@uniba.it; 4 Pediatric Gastroenterology and Hepatology Unit, Department of Interdisciplinary Medicine, Children’s Hospital—Giovanni XXIII, University of Bari Aldo Moro, 70121 Bari, Italy; fernandacristofori@gmail.com (F.C.); rfrancavilla@gmail.com (R.F.)

**Keywords:** autism spectrum disorder (ASD), proton nuclear magnetic resonance (1H-NMR) spectroscopy, metabolomics, leaky gut, gut microbial dysbiosis, intestinal mucosal permeability, lactulose:mannitol test

## Abstract

Several metabolomics-based studies have provided evidence that autistic subjects might share metabolic abnormalities with gut microbiota dysbiosis and alterations in gut mucosal permeability. Our aims were to explore the most relevant metabolic perturbations in a group of autistic children, compared with their healthy siblings, and to investigate whether the increased intestinal permeability may be mirrored by specific metabolic perturbations. We enrolled 13 autistic children and 14 unaffected siblings aged 2–12 years; the evaluation of the intestinal permeability was estimated by the lactulose:mannitol test. The urine metabolome was investigated by proton nuclear magnetic resonance (1H-NMR) spectroscopy. The lactulose:mannitol test unveiled two autistic children with altered intestinal permeability. Nine metabolites significantly discriminated the urine metabolome of autistic children from that of their unaffected siblings; however, in the autistic children with increased permeability, four additional metabolites—namely, fucose, phenylacetylglycine, nicotinurate, and 1-methyl-nicotinamide, strongly discriminated their urine metabolome from that of the remaining autistic children. Our preliminary data suggest the presence of a specific urine metabolic profile associated with the increase in intestinal permeability.

## 1. Introduction

Autism spectrum disorder (ASD) is a group of pervasive neurodevelopmental conditions affecting more males than females in a ratio close to 3:1 [1]; ASD is characterized by complex, heterogeneous clinical phenotypes frequently associated with medical comorbidities [2,3]. In particular, gastrointestinal disorders such as diarrhea, constipation, abdominal pain [4], sleep disturbances [5], and epilepsy [6] are frequently observed in children and adults with ASD. A large portion of individuals with ASD exhibits gut dysbiosis, suggesting a close relationship between specific perturbations in the gut bacterial community with the etiopathogenesis and the severity of autism [7,8]. The overgrowth of specific microbial species and genera, together with the increased biosynthesis of harmful microbial metabolites, severely alters the integration of gut microbiota with the central nervous system (CNS), the immune system, and the host metabolism [9]. Over the last few decades, the prevalence of ASD has dramatically increased; in the United States of America (USA), the ASD prevalence is approximately 1.85% [10,11]. Several metabolomics-based studies have demonstrated that autistic subjects share metabolic abnormalities linked with amino acid and purine metabolisms, energy production, oxidative stress, and gut microbiota fermentation of nutrients and toxicants [12]. Thus, the detection of the metabolic fingerprint in individuals with ASD could improve the early diagnosis of the disease, especially under two years of age, allowing earlier effective therapeutic interventions. We aimed to explore the most relevant metabolic perturbations in a group of ASD children, compared with their healthy siblings, and to investigate whether alterations of the intestinal permeability in autistic children may induce specific perturbations in the urine metabolome.

## 2. Results

Overall, 27 children were enrolled in this study, 13 with ASD and 14 unaffected siblings (US). Based on the preliminary principal component analysis (PCA), two US (identified as #2 and #21) were classified as outliers and ruled out; both were affected by severe dyslexia. As a result, 12 US were finally included in this study. The lactulose:mannitol test unveiled two ASD children with increased intestinal permeability (child #7 and child #10); compared with the median value of test results in ASD children with normal intestinal permeability, child #7 had a difference of +156%, and child #10 a difference of +195%). Demographic data are summarized in Table 1.

Compared with the group of ASD children with normal intestinal permeability, the two children with increased intestinal permeability showed several differences in demographic data. Child #7 was born vaginally, while child #10 by cesarean section; their respective mothers had no previous abortion, and both children did not exhibit constipation. Variations in other demographic data are reported in Table 2.

**Figure 1:**
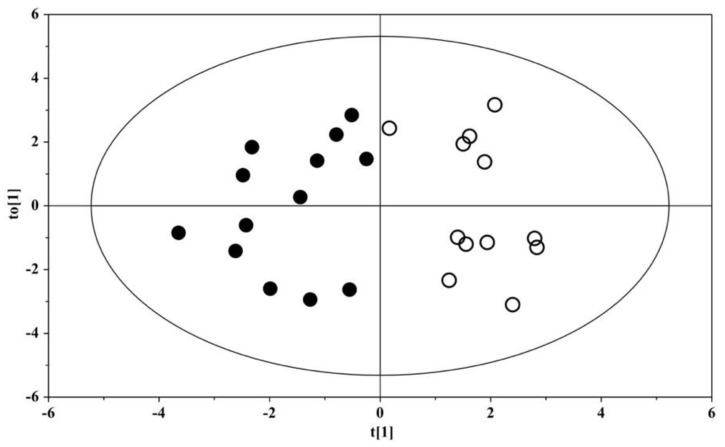
OPLS-DA scores plots of ^1^H NMR spectra of urine samples. Solid (black) dots: ASD children (*n* = 13); open dots: US (*n* = 12).

A model based on the orthogonal projection to latent structure discriminant analysis (OPLS-DA) was built for the dataset; the OPLS-DA scores plot evidenced a clear separation between the urine metabolome of ASD children and that of their US (Figure 1). The OPLS-DA model was established with one predictive and one orthogonal component, showing significant R^2^X, R^2^Y, and Q^2^ (Table 3); thus, the model was considered robust and statistically significant.

The validity of the OPLS-DA model was evaluated through a permutation test (Figure S1). Univariate and multivariate statistical analysis identified nine metabolites as those best-discriminating ASD children from their US; in detail, in ASD children, urine levels of 2-hydroxybutyrate, asparagine, hippurate, glutamate, tryptophan, and tyrosine were significantly higher than those in the US, while histidine, isocitrate, and succinylacetone were significantly lower (Table 4).

**Figure 2:**
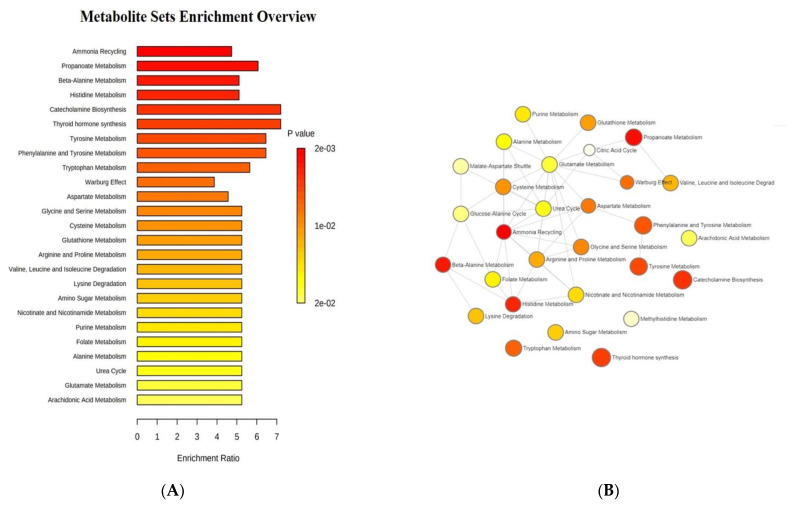
Enrichment analysis: (**A**) list of the most significantly discriminant pathways between ASD children and their unaffected siblings; (**B**) network analysis illustrating the most significant relationships between perturbed biochemical pathways in our group of autistic children. Metabolic pathways are represented as circles according to their scores from enrichment (vertical axis) and topology analyses (pathway impact, horizontal axis). Darker circle colors indicate more significant changes of metabolites in the corresponding pathway. The size of the circle corresponds to the pathway impact score and is correlated with the centrality of the involved metabolites.

The enrichment analysis unveiled that catecholamine and thyroid hormone biosynthesis, phenylalanine and tyrosine metabolism, as well as β-alanine, tryptophan, and histidine metabolisms, were the most significantly discriminant pathways between ASD children and their unaffected siblings, as reported in Figure 2A. The network analysis demonstrated a close relationship between various metabolic pathways, for example, between histidine metabolism and β-alanine, nicotinate, and nicotinamide metabolisms, as well as between the metabolism of tyrosine and that of glutamate via aspartate (Figure 2B).

**Figure 3:**
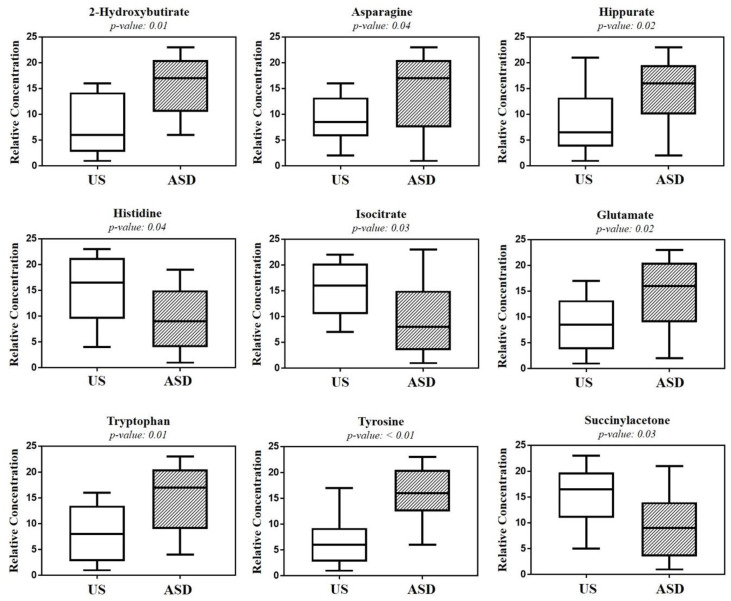
Box-and-whisker plots show the progressive change in the urine metabolite levels of autistic children (ASD), compared with their unaffected siblings (US). Statistical significance was determined by the Mann–Whitney U test after the Benjamini–Hochberg adjustment; *p* < 0.05 was considered statistically significant.

**Figure 4:**
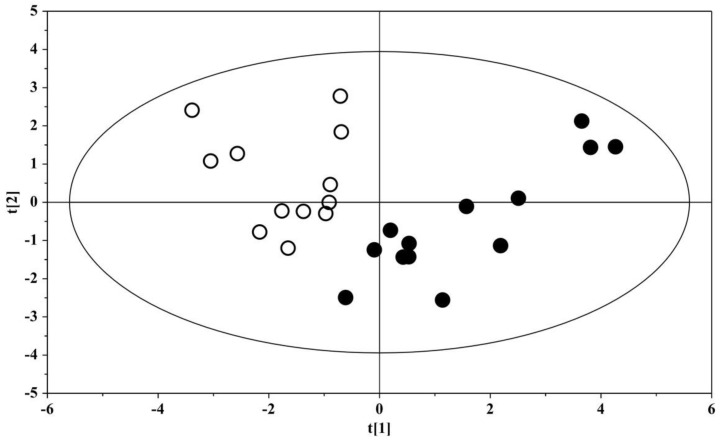
PCA scores plot built with the nine identified significant metabolites: solid (black) dots, ASD children (*n* = 13); open dots, US (*n* = 12).

The box-and-whisker plot (Figure 3) represents differences between groups in urine metabolites concentration. The receiver operating characteristic (ROC) plot was built by combining the nine discriminant metabolites (Figure S2). The area under the curve (AUC) was 0.737 (95% CI: 0.535–0.939), indicating the good predictive accuracy of the model. Subsequently, a new PCA model was prepared by using only the nine discriminant metabolites previously identified (Figure 4).

The PCA plot shows the projection of the samples on the plane formed by the first two PCs that explain 73.0% of the total variance; a clear separation between the urine metabolome of ASD subjects and that of the US was evident, suggesting the key role of the nine discriminant metabolites for the group separation. The association between metabolites abundance and the Autism Diagnostic Observation Schedule, Second Edition, with the calibrated severity score (ADOS-2 CSS), demonstrated a significant correlation for hippurate (r = −0.678 (95% C.I. −0.898 to −0.186), *p* = 0.0129) obtained by applying the Spearman’s correlation test (Figure S3).

**Figure 5:**
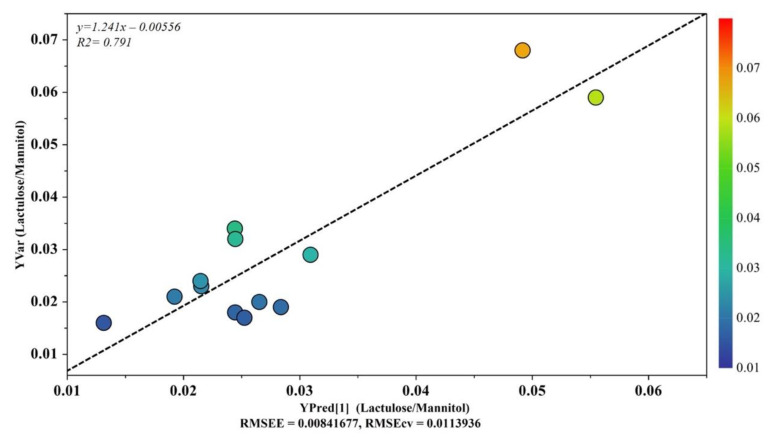
OPLS plot illustrating the relationship between the metabolic profile and the intestinal permeability scores. The horizontal axis represents the observed values, and the vertical axis represents the predicted values.

OPLS analysis was applied to ASD samples to evaluate the potential relationship between the urine metabolome (X variable) and the intestinal permeability test (Y variable). The OPLS model was established with one predictive and one orthogonal component. The OPLS model clearly indicated that the urine metabolic profile had a good fit and prediction ability for the intestinal permeability value, with R^2^ = 0.791 (Figure 5). The validity of the OPLS model was evaluated through a permutation test using 400 times. The model was statistically valid, with R^2^X = 0.359, R^2^Y = 0.662, and Q^2^ = 0.478 (Table 3).

**Figure 6:**
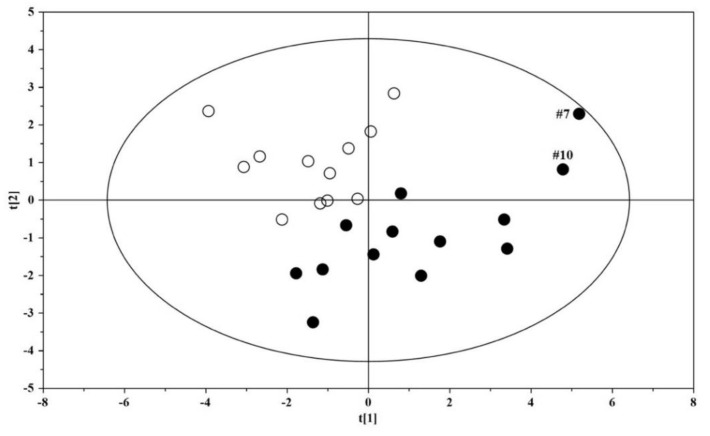
PCA scores plot built with the thirteen identified significant metabolites: solid (black) dots, ASD children (*n* = 13); open dots: US (*n* = 12).

Based on results that emerged from the OPLS model (Figure 5), we aimed to investigate any further metabolic change induced by the altered intestinal permeability. Discriminant metabolites were selected by analyzing the loadings plots based on color codes; only metabolites characterized by *p*(corr) > 0.6. were quantified. As a result, we found that fucose, phenylacetylglycine, nicotinurate, and 1-methyl-nicotinamide were significantly increased in ASD children with altered gut permeability but not in the remaining ASD children. Thus, a new PCA was built with the nine metabolites previously identified (Figure 3), and the four discriminant metabolites in ASD children with altered gut permeability. The resulting PCA model showed the strong influence of the overexpression of these metabolites in the urine metabolome, suggesting a candidate metabolic fingerprint of the altered gut permeability (Figure 6).

## 3. Discussion

The urine metabolome of children with ASD is clearly distinguished from that of their US (Figure 1), shaping a homogeneous set marked by a significant increase in six metabolites and a decrease in histidine, isocitrate, and succinylacetone (Figure 3 and Table 4). Our results demonstrate the presence of perturbations in pathways involved in the metabolism of tyrosine, asparagine, and tryptophan (Figure 2A), confirming previous findings published elsewhere [13,14]. In particular, the resulting increase in tryptophan in the urine of ASD children confirms our previous findings obtained on different patient cohorts by using both gas chromatography–mass spectrometry (GC–MS) [12,15] and proton nuclear magnetic resonance (1H-NMR) spectroscopy [16]. There is a broad agreement on the assumption that the vagal nerve, microbial end-products, and tryptophan metabolites are the mainstay of the complex communication network between the gut and the CNS—namely, the gut–brain axis [17]. Specifically, perturbations in the metabolism of tryptophan, and in particular in the biosynthesis of the catecholamine precursors, imply a considerable impact on the CNS, and in particular on the development and severity of autism core symptoms. Specifically, aromatic amino acids can be metabolized by various gut microbial strains, including *Clostridium sporogenes* and *C. botulinum* [18]; various intermediates originating from the bacterial metabolism of tryptophan, tyrosine, and phenylalanine may alter the gut–brain axis and can act as neurotoxic molecules after passing the blood–brain barrier [19]. Indeed, in autistic subjects, several end-products derived from the microbial metabolism of amino acids, lipids, and carbohydrates, such as propionic acid and *p*-cresol, have impacts on the biosynthesis of neurotransmitters and synaptic plasticity and memory formation in the brain.

**Figure 7:**
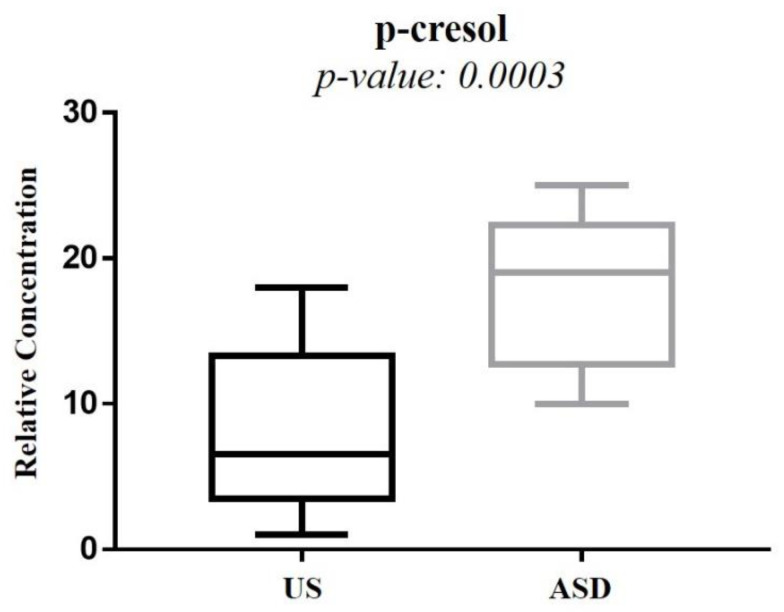
Box plot comparing the *p*-cresol concentration between autistic children and their unaffected siblings.

The significant increase in tyrosine in ASD children was of particular interest, given that we found the absence of any overlap between the interquartile range of tyrosine in ASD children and that in the US (Figure 3). Tyrosine catabolism involves five enzymatic steps, leading to the conversion of tyrosine to fumarate and acetoacetate; therefore, tyrosine can be considered both a glucogenic and ketogenic amino acid [20]. Several previous studies found a significant increase in tyrosine in ASD, both in urine [15,21,22] and blood [23]. Conversely, other studies reported a decrease in tyrosine in the urine [24,25,26,27] and plasma [28] of ASD children. Interestingly, in stools of autistic children with gastrointestinal (GI) disorders, tyrosine abundance was significantly lower than in autistic children without GI disorders; similarly, tyrosine was significantly reduced in children with high ADOS-2 CSS scores, corresponding to severe autistic core symptoms [29]. The dietary intake and the hydroxylation of phenylalanine are the main sources of tyrosine. Indubitably, the diet of autistic children (especially those with food selectivity) may originate, at least partially, controversial results from the literature, as highlighted elsewhere [21]. The observed increase in tyrosine concentration in the urine of our autistic children suggested an in-depth search on alterations in tyrosine catabolites. As expected, we found a significant increase in *p*-cresol in the urine of autistic children, compared with their US (Figure 7). *Para*-cresol (4-methylphenol) is an organic aromatic compound mainly derived from the microbially mediated decarboxylation of *p*-hydroxyphenylacetate, an intermediate of the tyrosine catabolism. Notably, high levels of *p*-hydroxyphenylacetate are directly related to gut bacterial overgrowth and dysbiosis with the prevalence of *Clostridium difficile* [30]. However, at least 55 bacteria strains have been identified as *p*-cresol producing bacteria, including *Clostridium difficile*, *Blautia hydrogenotrophica*, and *Romboutsia lituseburensis* belonging to the *Clostridia* class (*Firmicutes* phylum), and *Olsenella uli* belonging to the *Coriobacteria* class (*Actinobacteria* phylum) [31]. In addition, *Pseudomonas stutzeri* produces *p*-cresol from toluene by expressing the enzyme toluene monooxygenase [32]. Our results are in line with data from the literature [33,34,35]; in fact, in individuals with ASD, gut dysbiosis, in conjunction with the gut mucosa inflammation and the slow intestinal transit, promotes an increase in *p*-cresol in urine and blood [36]. Less than 1% of total *p*-cresol is present in free form; approximately 95% of *p*-cresol is metabolized to *p*-cresol sulfate, prevalently in the colon and liver, and the remaining 3–4% is metabolized to *p*-cresol glucuronide in the liver only [37]. Beyond the well-known toxicity of *p*-cresol and that of its end-products *p*-cresol sulfate and *p*-cresol glucuronide [38], a recent study on animal models found that *p*-cresol selectively induces ASD core behavioral symptoms [39]. In individuals with ASD, *p*-cresol abundance positively correlates with the severity of the disease [40]; a possible mechanism of *p*-cresol toxicity is the inhibition of the enzyme dopamine β-hydroxylase, leading to an imbalance of neurotransmitters and playing a primary role in the accumulation of dopamine [41]. In our cohort of autistic children, a further metabolic perturbation confirming gut dysbiosis with the overgrowth of *C. difficile* is the significant increase in urine hippurate, the glycine conjugate of benzoic acid [42]. The increase in 2-Hydroxybutyrate can be associated with oxidative stress and the increased demand for energy in our ASD children. Only two previous studies found the increase in 2-Hydroxybutyrate in the urine of autistic subjects [21,27]; this short-chain carboxylic acid, also known as α-hydroxybutyrate, is involved in the propanoic acid metabolism and can be induced by repetitive dietary patterns with inadequate nutrient intake, as in ASD children with food selectivity [43]. In our autistic children, urine glutamate was significantly higher than in their US. In fact, results regarding urine and blood glutamate concentration in autism are controversial. Two studies found high levels of glutamate in the urine of autistic subjects, compared with neurotypical (NT) individuals [27,41], while three found the opposite [13,24,44]. A study reported no significant difference in urine glutamate levels between autistic subjects and NT individuals [26]. Similar discrepancies are detectable in studies on blood (plasma/serum) glutamate levels in autism [45,46,47,48]. Theoretically, the recently confirmed decreased availability of pyridoxal-5-phosphate, the active form of vitamin B6, in ASD [41] largely supports the strength of studies reporting the increase in glutamate in autism. Indeed, P5P is a basic cofactor of the enzyme glutamate decarboxylase catalyzing the biotransformation of glutamate into GABA [49]; thus, the lack of P5P promotes glutamate accumulation. It is reasonable to argue that discrepancies between studies on glutamate concentration in ASD may be due to the influence of chemical–physical factors, such as the pH of biofluids. Changes in pH induce significant changes in glutamate concentration [50]; moreover, pH can impact glutamate and glutamine metabolism in the kidney, liver, and brain. The observed depletion of isocitrate and histidine in our autistic children is associated with mitochondrial impairment and oxidative stress, respectively [51,52], confirming the association between metabolic perturbations and autism.

As reported in Figure 5, the OPLS plot clearly showed that the urine metabolome of two ASD children was far from the cluster grouping the other ASD children; interestingly, the two children (#7 and #10) had an increased gut permeability, also called leaky gut. Indeed, the increased intestinal permeability was reflected by the presence of four discriminant metabolites—namely, fucose, phenylacetylglycine, nicotinurate, and 1-methyl-nicotinamide. Based on this finding, we decided to include these additional metabolites in our PCA model previously based on nine significant metabolites. Surprisingly, we observed that the two autistic children with increased gut permeability were clearly separated from the group of the remaining autistic children (Figure 6). Various factors may contribute to an increase in intestinal permeability, including altered physical barriers, gut dysbiosis, and diet. In autism, the frequent appearance of gastrointestinal comorbidities, such as chronic diarrhea, constipation, irritable bowel syndrome, and gastroesophageal reflux, originates from a progressive vicious circle involving gut dysbiosis, food selectivity (not always), gut mucosa inflammation, and leaky gut. As a result, microbial metabolites and toxins, cytokines, immunomodulators, and various molecular components of the bacterial membrane translocate from the intestinal lumen to the systemic circulation, leading to the impairment of the gut–brain integrity and function [53]. Fucose is crucial for maintaining a balanced gut ecosystem; perturbations in host fucosylation induce alterations in the immune functions and negatively impact the anti-inflammatory role of fucose [54]. Fucose is an abundant constituent of the mucus layer lining the mucosal epithelium of the gastrointestinal tract [55,56]. Thus, it is reasonable to argue that the increase in urinary fucose concentration observed in our ASD children, together with the increased gut permeability, may reflect the disruption of the gut mucosa with the release into circulation of this monosaccharide. Moreover, commensal bacteria, including *Bifidobacteria* spp. and *Bacteroides* spp., utilize fucose as a component of the bacterial outer membrane or as a carbon source for energy requirement. Consequently, changes in fucose concentration are closely related to differences in gut microbial composition marked by the overgrowth of pathogens, such as *Clostridium difficile*, and the reduction of commensals, for example, *Bifidobacteria* spp., typically recognizable in ASD [57]. The significant increase in urinary phenylacetylglycine in our ASD children with increased gut permeability may be related to the accumulation of phenylalanine, promoted by the microbial biosynthesis of aromatic amino acids via the shikimate pathway [58]. The conjugation of phenyl acetyl coenzyme A (CoA) with glycine originates phenylacetylglycine [59]; phenylacetate is the precursor for phenyl acetyl–CoA and can be synthesized by oxidation of phenyl-containing fatty acids. However, phenylacetate is also produced either by the degradation of phenylalanine to phenylethylamine and phenylacetaldehyde or via phenylpyruvate [60]. Phenylacetylglycine is a biomarker of phospholipidosis [61]; in a previously published study on 117 autistic children aged 2–18 years, the 1H NMR spectrum of urine analysis revealed a slight increase in phenylacetylglycine, compared with controls [62]. Currently, the role of phenylacetylglycine in ASD remains unclear; however, a recent study confirmed the close association between phenylacetylglycine and gut dysbiosis [63]. Briefly, six-week diet supplementation with a prebiotic (galactooligosaccharide) in a group of autistic children whose diet was not restricted and an exclusion diet (mainly gluten and casein-free) led to considerable changes not only in gut microbiota composition, including the increase in *Bifidobacterium* spp. but, even in the urine metabolome, with the significant reduction in phenylacetylglycine. High levels of urinary nicotinurate and 1-methyl-nicotinamide in autistic children with increased gut permeability unveils perturbations in the tryptophan-nicotinic acid metabolic pathway. Previous studies reported the increase in urinary nicotinamide by-products deriving from conjugation of nicotinic acid to glycine (nicotinurate) or from nicotinamide methylation (1-methyl-nicotinamide) in autistic children, compared with neurotypical individuals [44,64]; similar results were also found in plasma and associated with a significant increase in tryptophan plasma levels [65]. The increased presence of these catabolites in the urine of autistic children indicates the shunt of the tryptophan metabolism from the serotonin biosynthesis to the formation of nicotinic acid. It was postulated that in ASD children, the high level of urinary 1-methyl-nicotinamide reflects an increased need for niacin, also known as vitamin B3 or vitamin PP [64]. Remarkably, 1-methyl-nicotinamide is considered a biomarker of gut dysbiosis [66].

### Limitations of the Study

Our study has several limitations. First, the number of enrolled ASD children and their US was relatively small; thus, we cannot create a definitive model for ASD prediction. Second, only two ASD children presented an increased intestinal permeability, assessed by the lactulose:mannitol test; therefore, our results should be considered preliminary, and they need to be confirmed by further studies enrolling an adequate number of participants. Nevertheless, even though the increased intestinal permeability was found in only two ASD children, our results are original and stimulate the research of metabolic signatures and candidate biomarkers associated with leaky gut. Third, we performed neither metagenomic nor a culture-based analysis of the gut microbial flora. This limitation may be partially overcome by the identity of bacterial metabolites with the gut microbiota [67].

## 4. Materials and Methods

### 4.1. Participants

ASD children and their US were recruited by the Child Neurological and Psychiatric Unit, University-Hospital, Bari (Italy). The local institutional review board approved the study protocol. Informed consent from a parent or legal guardian was obtained for each participant. Diagnosis of ASD was established following the *Diagnostic and Statistical Manual of Mental Disorders* 5th Edition DSM-5 criteria [68]. Autistic children with other neurological disorders of known etiology, severe head injury, chronic gastrointestinal disorders and treated with any pharmacological therapy, at least in the month preceding the start of the study, were excluded.

### 4.2. Primary Behavioral Outcome Measures in Autistic Children

In ASD children, the severity of core deficits was evaluated by the Autism Diagnostic Observation Schedule, Second Edition, with the calibrated severity score (ADOS-2 CSS), performed by a licensed clinician. [69,70] The intestinal permeability was assessed by the non-invasive lactulose:mannitol test, based on the oral administration of a dose of both sugars, followed by a timed urine collection [71]. Mannitol (monosaccharide) and lactulose (disaccharide) have different molecular weights (182 Da and 342 Da, respectively) and different molecular diameters (6.5 Å and 9.5 Å, respectively). After ingestion, mannitol (monosaccharide) and lactulose (disaccharide) are passively absorbed through the gut mucosa and metabolized only in a small amount; therefore, they are excreted almost completely unchanged in the urine in proportion to the quantities absorbed. Mannitol is a marker of transcellular uptake, while lactulose is a marker of gut mucosa integrity [72]. Increased lactulose to mannitol ratio is an indicator of intestinal barrier dysfunction. For the lactulose:mannitol test, we followed the procedure previously reported elsewhere [73]. All the subjects did not assume any nonsteroidal anti-inflammatory drug.

### 4.3. Sample Collection, Storage, and Preparation

First-morning urine samples were collected within sterile bags and delivered to the Child Neurological and Psychiatric Unit, University-Hospital, Bari (Italy), before the end of that morning. Briefly, 800 μL of urine sample were transferred into cryo-vials, and an 8 μL 1% aqueous solution of NaN3 was added to inhibit bacteria growth; then, samples were frozen and stored at −80 °C until analysis. Before analysis, samples were centrifuged for 10 min at 4 °C at 12,000× *g* to remove solid particles. Then, 630 μL of the supernatant were mixed with 70 μL of potassium phosphate buffer in D_2_O (1.5 M, pH 7.4) containing sodium 3-trimethylsilyl-propionate-2,2,3,3,-d4 (TSP) as an internal standard (98 atom% D, Sigma-Aldrich, Milan, Italy). Finally, 650 μL were transferred to 5 mm NMR glass tubes for 1H-NMR analysis.

### 4.4. Proton Nuclear Magnetic Resonance (1H-NMR) Spectroscopy Analysis

^1^H-NMR analysis was carried out using a Varian UNITY INOVA 500 spectrometer operating at 499.839 MHz for proton and equipped with a 5 mm double resonance probe (Agilent Technologies, Santa Clara, CA, USA). One-dimensional proton NMR spectra were obtained by using a 1D Nuclear Overhauser Enhancement Spectroscopy (NOESY) standard pulse sequence to suppress water signals with a relaxation delay of 3 s. For each sample, 256 free induction decays (FIDs) were collected into 64K data points with a spectral width of 6000 Hz spectral with a 90° pulse, an acquisition time of 2 s, and a mixing time of 100 ms. The FIDs were weighted by an exponential function with a 0.5 Hz line-broadening factor prior to Fourier transformation.

### 4.5. Data Preprocessing

NMR spectra were phased and baseline corrected using an Advanced Chemistry Development (ACD) lab (Toronto, ON, Canada) Processor Academic Edition (Advanced Chemistry Development, 1 December 2010) and chemical shifts referenced internally to trisodium phosphate (TSP) at δ = 0.0 ppm. The spectral region comprising the signal of residual water and urea (4.7–6.5 ppm) was removed. The final spectral regions were between 0.5–4.7 ppm and 6.5–9.5 ppm. The ACD Labs intelligent bucketing method was used for spectral integration [74]. A 0.04 ppm bucket width was defined with an allowed 50% looseness, resulting in buckets that ranged between 0.02 and 0.06 ppm in width. The degree of looseness allows the bucket width to vary over a particular value from the set bucket value. The intelligent bucket method identifies local minima in the spectra and sets the buckets accordingly. In this manner, a peak is integrated into one bucket, although there may be minor chemical shift differences due to pH, for instance. The area of bucketed regions was normalized by using median fold change (MFC) normalization, largely preferred to total sum normalization when studying urine samples [75]. Finally, the spectral data were imported for multivariate statistical analysis into the SIMCA software (Version 15.0, Sartorius Stedim Biotech, Umea, Sweden). All imported data were then pre-processed using Pareto scaling by weighting each integral region or variable by (1/Sk) ½, where Sk represents the variable’s standard deviation. This procedure increased the representation of lower concentration metabolites in the resultant data models while minimizing the noise contribution.

### 4.6. Statistical Analysis

Variables were quantified using the Chenomx NMR Suite 7.1 (Chenomx Inc., Edmonton, AB, Canada), an integrated set of tools for identifying and quantifying metabolites in NMR spectra [76]. Chenomx NMR Suite is equipped with reference libraries containing numerous pH-sensitive compound models that are identical to the spectra of pure compounds obtained under similar experimental conditions. GraphPad Prism software (version 7.01, GraphPad Software, Inc., San Diego, CA, USA) was used to perform the univariate statistical analysis. Statistical significance was assessed by using the Mann–Whitney U test; *p* < 0.05 was considered statistically significant. The Benjamini–Hochberg adjustment was subsequently applied to *p*-values, to acquire the level of significance for multiple testing [77]. To further evaluate the diagnostic robustness of potential biomarkers, receiver operating characteristic (ROC) was carried out. The GraphPad Prism software generated ROC curves and calculated sensitivity, specificity, and area under the ROC curve (AUC). Finally, Spearman’s correlation test compared the metabolite abundance and the clinical ADOS score.

Multivariate statistical analysis was based on principal component analysis (PCA), orthogonal partial least-squares discriminant analysis (OPLS-DA), and orthogonal partial least squares (OPLS). PCA evaluated the homogeneity of the samples for ASD subjects and their US and identified any possible trend and/or outlier. OPLS-DA was used to reduce model complexity and to highlight samples discrimination better. The model’s goodness was evaluated using 7-fold cross-validation and a “permutation test” (400 times). The permutation test was calculated by randomizing the Y matrix (class assignment or continuous variables), while the X matrix (peak intensity in NMR spectra) was kept constant. The permutation plot then displays the correlation coefficient between the original y-variable and the permuted y variable on the *x* axis versus the cumulative R^2^ and Q^2^ on the *y* axis and draws the regression line. The intercept is a measure of the overfit; a Q^2^Y intercept value less than 0.05 indicates a valid model. The estimated predictive power of the models was expressed by R^2^Y and Q^2^Y, which represent the fraction of the variation of Y variable and the predicted fraction of the variation of Y variable, respectively. A good prediction model is achieved when Q^2^ > 0.5. In order to highlight potential metabolites that mainly contributed to group separation, an S plot for the OPLS-DA model was created. The S plot for NMR spectroscopy data combines the covariance (peak height) and correlation (color code) for the model variables displaying both in a single graph. OPLS regression analysis was used to investigate the relationship between intestinal permeability and normalized metabolomics profile of the ASD subjects. The quality of the OPLS model was evaluated using a 7-fold cross-validation and permutation test (400 times).

## 5. Conclusions

In conclusion, our study provides preliminary insights into the metabolic fingerprint of autistic children with increased intestinal permeability; in particular, the presence and the severity of the increased intestinal permeability could be associated with alterations in the urinary concentration of specific metabolites, promoting further studies utilizing a targeted approach for the identification of candidate biomarkers of increased intestinal permeability. Finally, results emerging from our study confirm the central role of tryptophan metabolism in autism.

## Figures and Tables

**Table 1:** Demographic data. Variables are expressed as median and (interquartile range), except the ratio male/female, vaginal birth/caesarian section, previous abortion, constipation, and social status (*n*).

Variable	ASD ^1^ Children (*n* = 13)	ASD Children Excluding Those with Altered IP ^2^ (*n* = 11)	US ^3^ (*n* = 12)
Male/Female (*n*)	10/3	8/3	8/4
Age (years)	8 (4–12)	8 (4–12)	9 (13–5)
Vaginal birth/Cesarean section (*n*)	7/6	6/5	5/7
Gestational age (weeks)	39 (37–40)	39 (37–40)	39 (38–40)
Birthweight (kg)	3.25 (3.0–3.45)	3.20 (2.99–3.35)	3.54 (3.05–3.76)
Birth height (cm)	50 (48–50)	50 (46.5–50.5)	51 (49.8–53.2)
Mother’s age (years)	35 (32–36)	35 (33–37)	32 (25.7–37.0)
Father’s age (years)	40 (41–34)	40 (33.5–41.5)	35 (30.2–41.2)
Parental age gap (years)	4 (2–5)	4 (2.5–6.0)	4 (2.0–5.5)
Previous abortion (*n*)	2	0	2
Constipation (*n*)	5	5	2
ADOS-2 CSS ^4^ (score)	10 (8–12)	9 (7.5–11)	-
Lactulose:mannitol ratio	0.023 (0.19–0.032)	0.021 (0.019–0.027)	0.023 (0.015–0.029)

^1^ ASD, autism spectrum disorder; ^2^ IP, intestinal permeability; ^3^ US, unaffected siblings; ^4^ ADOS-2 CSS, Autism Diagnostic Observation Schedule, Second Edition, with the calibrated severity score.

**Table 2:** Variations in demographics of the ASD children with increased intestinal permeability compared to median values in the remaining ASD children and in their US.

Variable	Child #7 (G.B., Male)		Child #10 (S.A., Male)	
	Variation from Median Value in ASD ^1^ Children	Variation from Median Value in US ^2^	Variation from Median Value in ASD ^1^ Children	Variation from Median Value in US ^2^
Age (years)	+2 (+25%)	+1 (+11.1%)	−3 (−37.5%)	−2 (−22.2%)
Gestational age, weeks (%)	−1 (−2.5%)	−1 (−2.5%)	0 (0%)	0 (0%)
Birthweight, kg (%)	+0.40 (+12.3%)	+0.11 (+3.1%)	+0.20 (+6.1%)	−0.09 (−2.5%)
Birth height, cm (%)	0 (0%)	−1 (−1.9%)	−1 (−2.0%)	−2 (−3.9%)
Mother’s age, years (%)	0 (0%)	+3 (+9.3%)	−3 (−9.3%)	0 (0%)
Father’s age, years (%)	−3 (−7.5%)	+2 (+5.7%)	−6 (−15%)	−1 (−2.8%)
Parental age gap, years (%)	−2 (−50%)	−2 (−50%)	−2 (−50%)	−2 (−50%)
ADOS-2 CSS ^3^, score (%)	−1 (−10%)	-	+9 (+90%)	-
Lactulose:mannitol ratio (%)	+0.036 (+156%)	+0.036 (+156%)	+0.045 (+195%)	+0.045 (+195%)

^1^ ASD, autism spectrum disorder; ^2^ US, unaffected siblings; ^3^ ADOS-2 CSS, Autism Diagnostic Observation Schedule, Second Edition, with the calibrated severity score.

**Table 3:** Statistical parameters of the OPLS-DA and OPLS models derived from the 1H NMR spectra of urine samples. ASD, autism spectrum disorder; US, unaffected siblings; OPLS-DA, orthogonal projection to latent structure discriminant analysis; 1H NMR proton nuclear magnetic resonance.

	OPLS-DA Model				Permutation (400 Times) *	
	Component ^a^	R^2^X Cum ^b^	R^2^Y Cum ^c^	Q^2^ Cum ^d^	R^2^ Intercept	Q^2^ Intercept
ASD vs. US	1P + 1O	0.229	0.801	0.504	0.394	−0.315
	**OPLS model**					
ASD children	1P + 1O	0.359	0.662	0.478	0.467	−0.331

^a^ The number of predictive and orthogonal components used to create the statistical models. ^b, c^ R^2^X and R^2^Y indicated the cumulative explained fraction of the X block and Y block variation for the extracted components. ^d^ Q^2^ indicates cumulative predicted fraction of the variation of the Y block for the extracted components. * Q^2^ intercept less than 0.05 indicates a valid model.

**Table 4:** Relative concentrations of discriminant metabolites in autistic children (ASD), compared with their unaffected siblings (US). Data are expressed as median and (interquartile range).

Metabolite (mM) ^a^	ASD	US	*p* ^b^	FC ^c^ (log10)
2-Hydroxybutyrate	5.24 (3.8–7.0)	3.05 (2.5–4.3)	0.01	0.778
Asparagine	5.12 (2.8–7.1)	3.18 (2.4–3.7)	0.04	0.686
Hippurate	5.79 (2.7–7.0)	2.07 (1.3–3.8)	0.02	1.482
Histidine	3.25 (1.6–5.0)	5.44 (3.5–7.9)	0.04	−0.739
Isocitrate	3.62 (2.6–4.4)	4.58 (3.8–6.2)	0.03	−0.337
Glutamate	5.35 (3.6–6.7)	3.48 (2.5–4.1)	0.02	0.618
Tryptophan	4.87 (3.4–7.2)	3.34 (2.2–4.2)	0.01	0.542
Tyrosine	4.75 (2.3–7.9)	3.03 (2.5–3.4)	<0.01	0.650
Succinylacetone	3.58 (2.6–4.5)	4.92 (3.8–5.8)	0.03	−0.458

^a^ For each sample, the relative concentration was obtained by normalizing the molar concentration of each metabolite to the total molar concentration of all nine metabolites. ^b^ Mann–Whitney U test after Benjamini–Hochberg adjustment was used; *p* < 0.05 was considered statistically significant. ^c^ Fold change (FC) between ASD children and their US; positive value refers to relatively higher metabolite concentrations, and a negative value to relatively lower metabolite concentration in ASD group, compared with their US.

## Data Availability

Data are available upon request to the corresponding author.

## Supplementary Materials

The following are available online at https://www.mdpi.com/article/10.3390/metabo12020104/s1, Figure S1: Validation plots of OPLS-DA model by using a permutation test. The horizontal axis shows the correlation between the permuted and actual data, while the vertical axis displays the cumulative values of R^2^ and Q^2^. The intercept gives an estimate of the overfitting phenomenon, Figure S2: Receiver operating characteristic (ROC) plot built by combining all significantly altered metabolites between ASD children and their US. Area under the curve (AUC) = 0.737, *p* = 0.04, Figure S3: Inverse Spearman’s correlation plot between urine hippurate abundance and the ADOS-2 CSS score in the group of autistic children (*n* = 13).
