# Supplementary material for: Alterations of the Intestinal Permeability are Reflected by Changes in the Urine Metabolome of Young Autistic Children: Preliminary Results
Source: Metabolites. 2022 Jan 23;12(2):104. doi: 10.3390/metabo12020104 (PMC8875518; doi:10.3390/metabo12020104)
Supplement: Supplementary file 1 [file metabolites-12-00104-s001.zip › metabolites-1547158-supplementary.pdf]

Alteration of the Intestinal Permeability are Reflected by Changes in the Urine Metabolome of Young Autistic Children

Supplementary materials

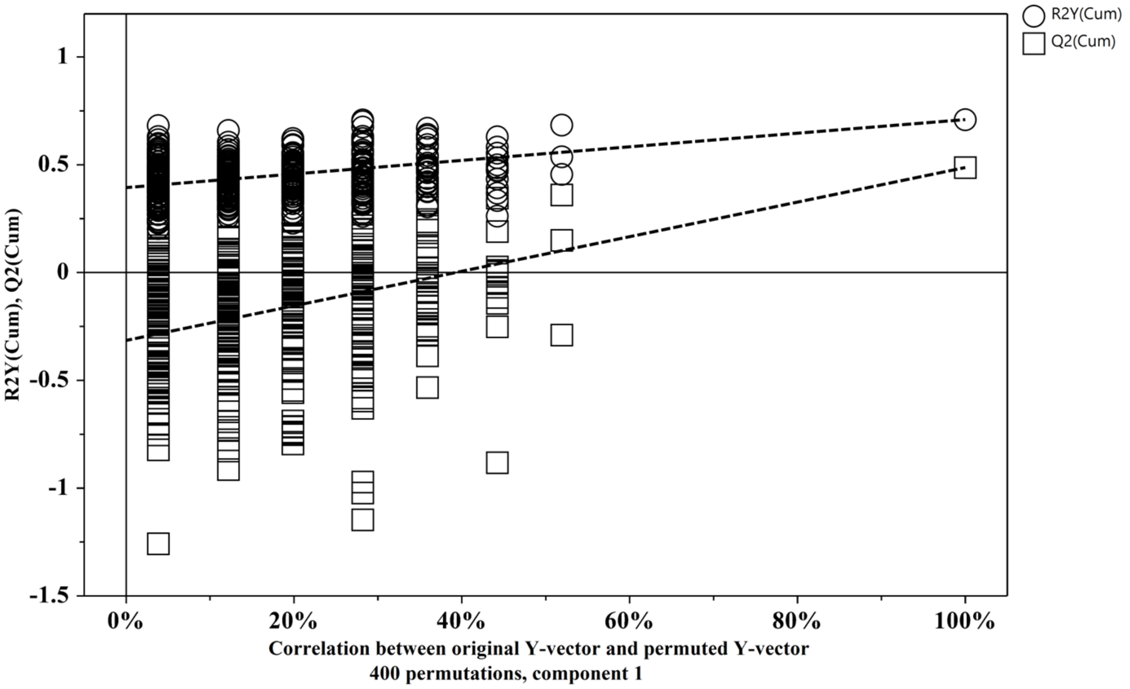

Figure S1. Validation plots of OPLS-DA model by using a permutation test. The horizontal axis shows the correlation between the permuted and actual data, while the vertical axis displays the cumulative values of R_2 and Q_2. The intercept gives an estimate of the overfitting phenomenon.

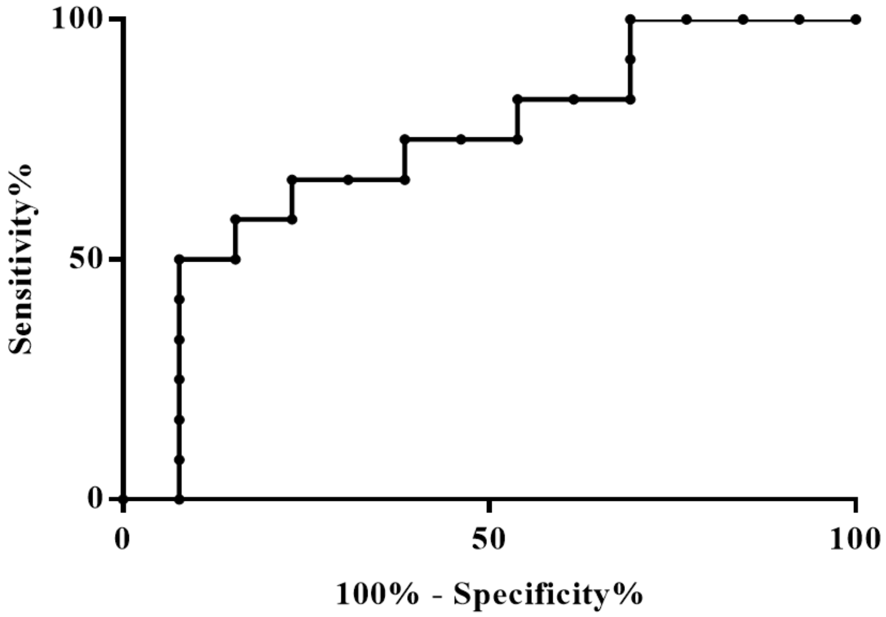

Figure S2. Receiver Operating Characteristic (ROC) plot built by combining all significantly altered metabolites between ASD children and their US. Area under the curve (AUC) = 0.737, p = 0.04

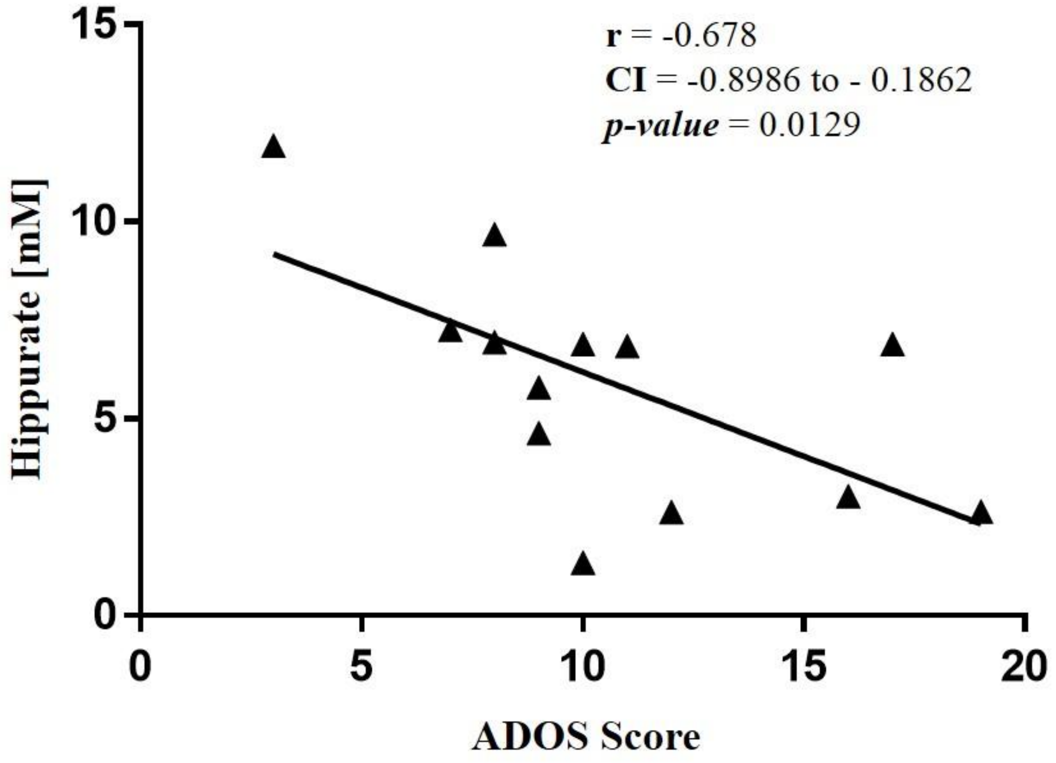

Figure S3. Inverse Spearman's correlation plot between urine hippurate abundance and the ADOS-2 CSS score in the group of autistic children (n=13)
